# Effects of negative workplace behavior on job insecurity and turnover intention in healthcare workers: roles of psychological resilience

**DOI:** 10.3389/fpubh.2025.1493964

**Published:** 2025-05-23

**Authors:** Jie Zou, Yanhong Yang, Ling Chen, Yin Bi, Na Li, Qin Luo, Jin Zhang

**Affiliations:** Hepatobiliary Pancreatic Cancer Center, Chongqing University Cancer Hospital, Chongqing, China

**Keywords:** negative workplace behavior, cross-sectional study, mediator, moderator, healthcare workers

## Abstract

**Introduction:**

Although negative workplace behavior as a key factor in healthcare staff turnover intention was well established, the mechanisms by which negative workplace behavior affects turnover intention are unclear. Extending the affective event theory, we aimed to (a) identify the interrelationships between negative workplace behavior, job insecurity, psychological resilience, and turnover intention in the healthcare setting and (b) clarify the mechanism among these variables.

**Methods:**

A cross-sectional survey was conducted in China from February to April 2023 utilizing a quota sampling method. The Chinese version of the negative behaviors in health care survey, the workplace insecurity scale, the 10-item Connor-Davidson Resilience Scale, and the turnover intention scale were used to investigate.

**Results:**

The survey resulted in 1,180 valid responses. Results were consistent with our hypothesized framework in which healthcare workers' turnover intention was significantly and positively influenced by negative workplace behavior (β = 0.251, *p* < 0.01) and job insecurity (β = 0.322, *p* < 0.01). Job insecurity partly mediated the association between negative workplace behavior and turnover intention, which were significantly moderated by psychological resilience (β = −0.041, *p* < 0.05).

**Conclusions:**

Negative workplace behavior is critical in turnover intention among healthcare workers. One important consideration for hospital administrators and health policymakers is creating a peaceful and harmonious workplace to reduce the risk of unfavorable workplace conduct and turnover intention toward healthcare personnel. An essential psychological resilience-improving program should be developed to reduce the damage of negative workplace behavior and job insecurity against healthcare workers.

## 1 Introduction

The shortage of healthcare workers is a serious and complex issue faced by the global healthcare system (1). The World Health Organization (WHO) noted that 12.9 million healthcare professionals will be short worldwide by 2035 (2). It has been observed that hospital nurses in various countries (such as Jordan, China) experience elevated levels of job burnout, heavy workloads, and a propensity to consider leaving their positions (3, 4). According to a survey from American Nurses Foundation indicated that 23% registered nurses intended to leave their position and 29% were considering leaving their current position within the next 6 months attributed to the pandemic, high job demands and burnout (5, 6). Turnover intention, defined as a conscious and deliberate willingness to leave the organization, is one of the major factors contributing to employee resignations and the strongest indicator that can predict an individual's turnover behavior (7). Studies confirmed that the high turnover rate of healthcare professionals increase costs and contribute to instability in the work environment owing to healthcare worker shortages (7, 8). The cost of employee turnover is thought to be associated with a minimum of 5% of the annual revenue loss (9). Research showing the high cost of turnover and recruitment reflects the importance of employment stability. Turnover intentions of healthcare workers may predict turnover behavior which deserves further attention and investigation.

### 1.1 Internal relationships between negative workplace behavior and turnover intention

A growing body of studies highlighted that one vital factor affecting the turnover intention of healthcare staff is the work environment (1). Workplace violence by patients or family members has received wide attention, but negative workplace behavior within the organization has been generally ignored. Negative workplace behavior involves psychological and physical hostility within the work environment that is pervasive and far-reaching for both the organization and its members (10). It can be divided into horizontal aggression between peers and vertical aggression between a leader and follower according to the directionality of the behavior (11). Due to the potential conflicts of interest and high-pressure work environments, negative behaviors within organizations can lead to endless stress and sustained pain for medical staff, which is no less harmful in the long run than bullying or violence from patients and family members (12). The unfavorable consequences of negative workplace behavior on turnover intention are well documented in past research (13). Previous studies have found that negative workplace behavior resulted in destroyed the harmonious atmosphere of the hospital (12), hindered healthcare workers' teamwork (14), and ultimately affected the safety of patients (15).

Although there is growing evidence that workplace incivility is an important predictor of various types of turnover intention, little is known about the association between negative workplace behavior and turnover intention among healthcare workers (16). Affective events theory (AET) can provide valuable insights for understanding the relationship between negative workplace behavior and turnover intention, which assumes that the work environment indirectly alters work attitudes by influencing work events (17). Negative workplace behavior from peers or leaders can impact healthcare professionals' working attitudes increasing the probability of turnover. Therefore, we attempt to explore the following research hypothesis:

**Hypothesis 1:** Negative workplace behavior has a positive association with healthcare workers' turnover intention.

### 1.2 The mediating role of job insecurity

Job insecurity refers to an individual's beliefs about the degree to which they fear losing their current job and the necessity of maintaining employment stability (18). The degree to which workers feel their employment is in jeopardy is reflected in their level of job insecurity (19). According to Hellgren et al. (20), there are two types of work insecurity: qualitative job insecurity, which refers to feelings of loss of important employment qualities, and quantitative job insecurity, which is based on subjective evaluations of the possibility of losing one's job. Previous study found that negative workplace behavior is a risk factor for job insecurity (21). Aisha Sarwar's study also revealed the link between job insecurity linking workplace bullying and nurse's deviant work behavior (13). Moreover, some scholars confirmed that workplace violence was positively related to healthcare workers' job insecurity. Few research, nevertheless, have looked at the possible mediating mechanisms that underlie the links between negative workplace behavior and turnover intention through job insecurity.

AET assumes that the work environment indirectly alters work attitudes by influencing work events, which subsequently trigger affective experiences (17). It offers a framework for investigating how job insecurity plays a significant role in mediating the relationship between negative workplace behavior and turnover intention (17). Events that occur to people in work environments and elicit feelings are referred to as work events (such as negative workplace behavior). Affective experiences are the feelings—whether favorable or unfavorable—that arise from work-related situations (such as job insecurity). Workplace attitudes (such as turnover intention) are directly impacted by the affective experience. Wu et al. (22) tested AET in a sample of 1,517 hospital nurses in China and found work environment was related to job satisfaction and intention to leave both directly and indirectly through two mediators: workplace violence and burnout. Therefore, we hypothesize:

**Hypothesis 2:** Job insecurity mediates the association between negative workplace behavior and turnover intention.

### 1.3 The moderating effect of psychological resilience

Psychological resilience is a psychological resource and stable personality trait that helps people deal with and overcome failure or adversity (23). A complicated and dynamic process, resilience involves nurses developing coping mechanisms to reduce psychological discomfort and problem-solving techniques to deal with occupational adversity (24). AET holds that personal traits can regulate the relationship between work events and affective experience (17). Healthcare professionals that possess psychological resilience are more likely to handle work-related stressors and experience personal development owing to they are more likely to mobilize internal modifications and utilize external resources (25). Furthermore, previous studies confirmed that the psychological health of emergency nurses may suffer from high rates of workplace violence, whereas resilience can help them lessen negative effects (26). Therefore, we hypothesize:

**Hypothesis 3:** Psychological resilience moderates the association between negative workplace behavior and job insecurity such that this positive relationship is stronger when the victim's psychological resilience is low.

**Figure 1:**
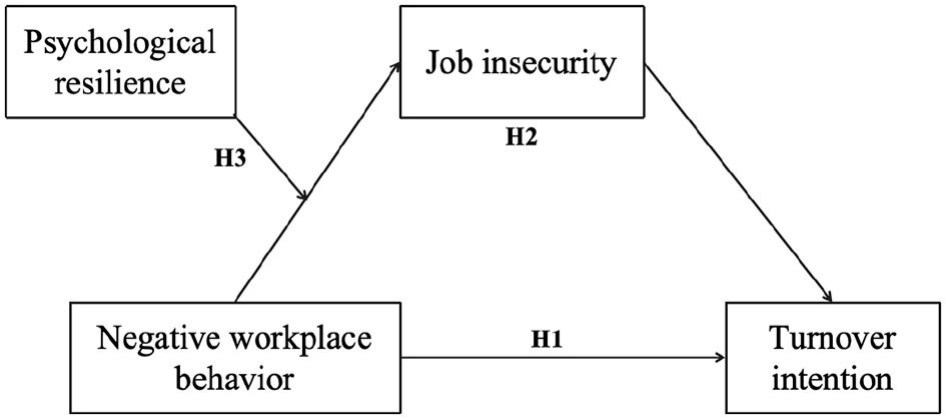
The conceptual model.

Based on the above literature review, the purpose of this study was to examine whether negative workplace behavior would have both direct and indirect effects on turnover intention through job insecurity and whether psychological resilience, as a positive resource, plays a moderating role in the process of negative workplace behaviors affecting job insecurity (Figure 1).

## 2 Methods

### 2.1 Participants and procedures

We used a quota sampling method to collect data, 1,108 healthcare workers were recruited between February and April 2023 from three tertiary hospitals in Hunan Province, China. According to data from the China Statistical Yearbook 2020, the ratio of medical personnel (doctors, nurses, pharmacists, technicians) was 1:1.52:0.12:0.13 (27). Therefore, we recruited 400 doctors, 608 nurses, 48 pharmacists, and 52 technicians. Inclusion criteria were as follows: (1) medical personnel with relevant qualifications; (2) working time of more than 3 months; (3) signed informed consent and were voluntarily willing to participate in this study. Trainees, interns, and those who could not cooperate in completing this study were excluded. The head of nursing at three hospitals sends other nurses a link to our self-administered questionnaire via Wenjuanxing (a professional online questionnaire platform in China, https://www.wjx.cn/). On the front page of self-administered questionnaires, they were then clearly informed of the goal and relevance of this study, and their participation was kindly requested.

The required sample size was calculated using the formula $n=Z_{\alpha /2}\pi \left(1-\pi \right)/\delta^{2}$. The type I error α was set as 0.05, the *Z*_α/2_ was set as 1.96, and the absolute error δ was set as 0.03. π = 50% was set for calculation in order to ensure sufficient size for cross-sectional study (28). A sample size of 961 was derived, and a minimum sample size of 1,057 was required given a 10% of invalid responses. Thus, 1,108individuals were recruited, which was sufficient to satisfy the cross-sectional survey's sample size requirements.

### 2.2 Variables and measurements

#### 2.2.1 General information questionnaire

The general information questionnaire was designed by looking through relevant research and the researchers' clinical expertise. Healthcare workers' demographic characteristics were collected. The information included age, gender, working years, marital status, education, and field.

#### 2.2.2 Negative workplace behavior

This study used the Chinese version of the NBHC questionnaire developed by Layne et al. (11). It was a specific tool designed to measure the negative workplace behaviors among healthcare professionals. The Chinese version of the scale included 23 items in 4 dimensions and 2 open-ended questions, including contributing factors (7 items), frequency of aggression (7 items), the severity of aggression (6 items), fear of retaliation (3 items), and 2 open-ended questions to describe medical staff's experience of negative behavior and suggestions for reducing negative behavior in the workplace. The dimensions of contributing factors and fear of retaliation were scored on a 4-point Likert scale ranging from 1 (“strongly disagree”) to 4 (“strongly agree”). The attack frequency dimension was scored on a 5-point Likert scale ranging from 1 (“never”) to 5 (“every day”). The attack severity dimension was scored on a 4-point Likert scale ranging from 1 (“not serious”) to 4 (“very serious”). The score of each dimension is the average score of all items in this dimension. The higher the score, the more serious the problem of negative behavior in the workplace. In this study, Cronbach's α of the total scale was 0.92.

#### 2.2.3 Job insecurity

The workplace insecurity scale (WIS) was developed by Hellgren et al. (20) and measures employees' fears about losing their jobs and their perceptions of threats to the quality of the employment relationship. It consists of 7 items scored on a 5-point Likert scale ranging from 1 (“strongly disagree”) to 5 (“strongly agree”), with total scores ranging from 7 to 35. A higher score represented higher levels of job insecurity experienced by the individual. The reliability of the Chinese version of the WIS was acceptable for the subscales (Cronbach's α = 0.949–0.903), and construct validity was good (29). The Cronbach's α was 0.96 for the total scale and 0.79–0.93 for five subscales in the present study.

#### 2.2.4 Psychological resilience

The 10-item Connor-Davidson Resilience Scale (CD-RISC-10) is a self-report measure designed for measuring resilience (30). The Chinese 10-item Connor-Davidson Resilience Scale was used in this study, which consists of 10 items scored on a 5-point Likert scale ranging from 0 (“not true at all”) to 4 (“true nearly all the time”) (31). The CD-RISC-10 generates total scores from 0 to 40 with higher scores indicating a greater ability to cope with adversity (32). This scale reported a good internal consistency (33). In this study, Cronbach's α of the total scale was 0.92.

#### 2.2.5 Turnover intention

Mobley developed the turnover intention scale in 1978 (34). The Chinese version of the turnover intention scale was used, which contains 4 items and uses the Likert-5 scale ranging from 1 (“completely inconsistent”) to 5 (“completely consistent”), with total scores ranging from 4 to 20 (35). A higher score indicated greater turnover intention. In this study, Cronbach's α of the total scale was 0.97.

### 2.3 Data analysis

Statistical analyses were performed using SPSS 22.0 and PROCESS. The correlation between the research variables was calculated using the Pearson correlation coefficient. The impact of this model was to be assessed by multiple linear regression analyses. Based on 5,000 bootstrapped samples, this study calculated the 95% bootstrap confidence intervals (CI). A significance level of *p* < 0.05 was used for all variables.

### 2.4 Ethics approval

The study complied with acknowledged ethical guidelines, as stated in the Declaration of Helsinki. The study received ethical approval from the Behavioral Medicine and Nursing Research Ethics Review Committee of Central South University (No. E E202217). Before starting data collection, electronic informed consent was obtained.

## 3 Results

### 3.1 Healthcare workers' demographic information

The majority of the participants (78.1%) were female, 26–30 years (31.9%). Most of the healthcare professionals had master's degrees (41.3%), followed by bachelor's degrees (26.2%), doctor's degrees (22.0%) and diplomas (10.5%). The present professional categories of the samples were nursing (54.9%), medicine (36.1%), pharmacy (4.3%), and technic (4.7%). The rest of the general data information is detailed in Table 1.

**Table 1:** Characteristics of the participants (*n* = 1,108).

**Characteristics**	**Categories**	***N***	**Percentage (%)**
Age	≤ 25	108	9.7
	26–30	353	31.9
	31–40	348	31.4
	41–50	261	23.5
	≥51	38	3.4
Gender	Male	243	21.9
	Female	865	78.1
Working years	< 1	198	17.8
	1–5	318	28.7
	6–10	329	29.7
	11–20	167	15.1
	>20	96	8.6
Marital status	Married	771	69.6
	Single	304	27.4
	Divorced	33	2.8)
Education	Diplomas	116	10.5
	Bachelor	290	26.2
	Master	458	41.3
	Ph.D.	244	22
Field	Doctors	400	36.1
	Nurses	608	54.9
	Pharmacists	48	4.3
	Technicians	52	4.7

### 3.2 Correlations between study variables

The descriptive statistical analysis results are shown in Table 2. Negative workplace behavior is significantly positively correlated with job insecurity (*r* = 0.317, *p* < 0.01). Job insecurity was positively correlated with turnover intention (*r* = 0.312, *p* < 0.01). There was a significant positive correlation between workplace negative behavior and turnover intention (*r* = 0.511, *p* < 0.01). This provides preliminary support for the hypothesis of this study.

**Table 2:** Descriptions and correlations of study variables.

**Variables**	**Mean ±SD**	**Range**	**1**	**2**	**3**	**4**
1. Negative workplace behavior	78.43 ± 10.92		1			
2. Job insecurity	23.20 ± 4.68	7–35	0.317^**^	1		
3. Resilience	27.94 ± 7.75	0–40	−0.278^**^	−0.377^**^	1	
4. Turnover intention	15.32 ± 4.28	5–25	0.511^**^	0.312^**^	−0.214^**^	1

^**^p < 0.01.

### 3.3 Multiple hierarchical linear regression models

**Figure 2:**
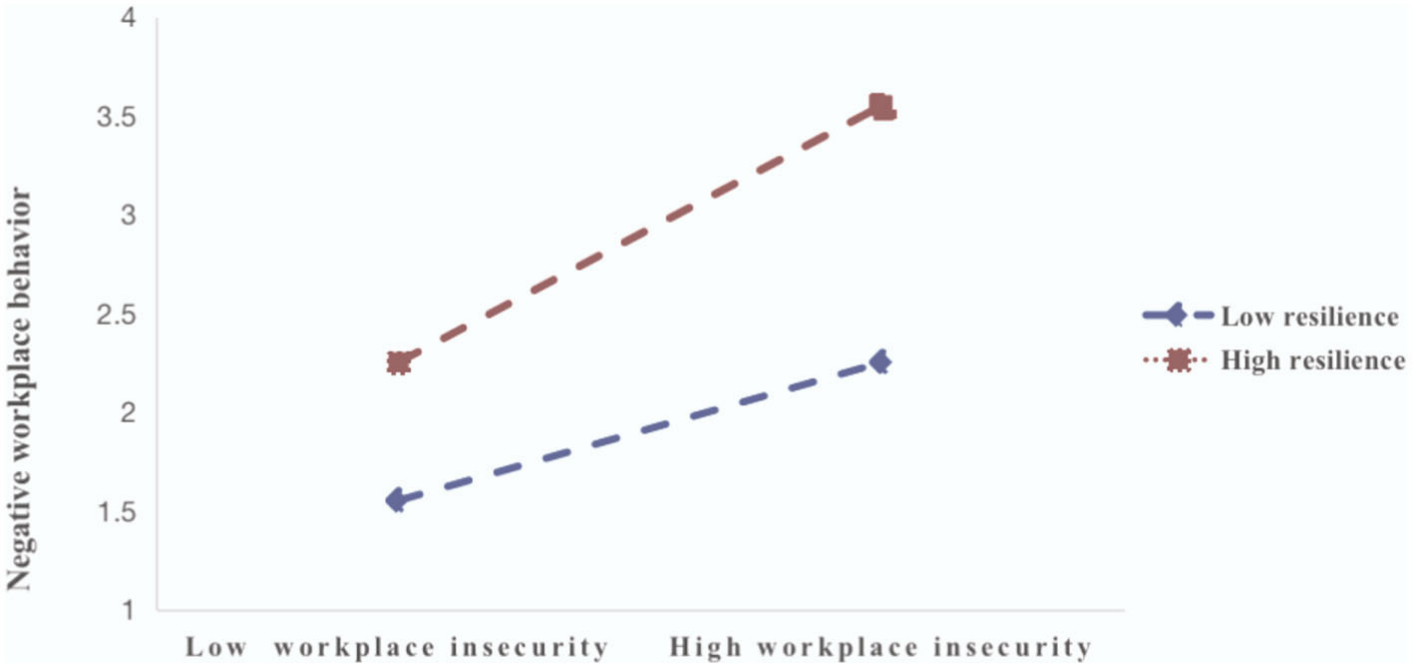
Moderated effect of resilience on the association between negative workplace behavior and job insecurity.

We performed multiple linear regression analyses to examine our hypotheses, testing the associations between negative workplace behavior, job insecurity, psychological resilience, and turnover intention, after adjusting for gender, age, marital status, education, and working year. Such variables were regarded as the control variables. Job insecurity was tested as a potential mediator of the association between negative workplace behavior and turnover intention. We tested the mediator via the PROCESS procedure for SPSS 22.0 by calculating bias-corrected 95% confidence using bootstrapping with *n* = 5,000 resamples. Negative workplace behavior was positively associated with job insecurity (β = 0.322, *p* < 0.01) and turnover intention (β = 0.251, *p* < 0.01); H1 and H2 were supported, as shown in Table 3. It showed that resilience significantly moderated the association between negative workplace behavior and work insecurity in Table 3 and Figure 2, so H3 was confirmed.

**Table 3:** Multiple hierarchical linear regression models.

**Variables**	**Job insecurity**	**Turnover intention**		
		**Hypothesis 1**	**Hypothesis 2**	**Hypothesis 3**
	**M1** ***(**β**)***	**M2** ***(**β**)***	**M3** ***(**β**)***	**M4** ***(**β**)***
**Control variables**				
Age	−0.628	−0.048	−0.117	−0.135
Gender	0.06	0.201	0.018	0.041
Working years	0.011	0.022	0.158	0.022
Marital status	0.933^**^	0.35	0.241	0.35
Education	−1.077^***^	−1.037	1.087	−1.213
Field	−0.107^**^	0.024	0.214	0.036
**Mediator**				
Job insecurity			0.256^**^	
**Moderator**				
Resilience				−0.038^**^
**Interaction**				
Negative workplace behavior^*^resilience				−0.041^**^
**Independent variable**				
Negative workplace behavior	0.322^**^	0.251^**^	0.152^**^	0.178^**^
F	13.352^***^	69.646^***^	7.236	6.802
R^2^	0.317	0.373	0.343	0.361
ΔR^2^	0.117	0.156	0.128	0.132

^**^p < 0.01, ^***^p < 0.001.

## 4 Discussion

The study results support the hypothesized model. Negative workplace behaviors positively influence Chinese healthcare professionals' turnover intention and job insecurity mediates their relationship. Furthermore, our research confirmed that psychological resilience moderates the association of negative workplace behaviors with job insecurity. Our research findings increase the understanding of how negative workplace behaviors influence turnover intention. A better understanding of this mechanism could provide a scientific basis for the prediction of nurse turnover intention and possible preventive measures.

As shown in our results, high negative workplace behaviors were related to increased turnover intention. This is consistent with previous research results, suggesting better work environment standards (e.g., true collaboration, authentic leadership) are associated with decreased turnover intention and improved employee satisfaction (36, 37). According to the theory of general aggression model, a person experiencing aggressiveness will go through three main stages: cognitive, physiological arousal, and emotional traits (38). This suggests that when healthcare workers experience negative workplace behaviors, they will engage in cognitive processing, which could result in emotional reactions like resentment and fear and eventually evolve into turnover intention (1). Preventing negative workplace behaviors is a potential strategy to reduce healthcare professionals' turnover intention. Therefore, it is recommended that hospitals set up psychological supervisory teams and form mental health departments for medical workers to reduce work stress and negative emotions by helping medical staff to channel their emotions. Our results add evidence to the importance of preventing negative workplace behaviors for reducing nurses' turnover intention.

Although the effect of negative workplace behaviors on turnover intention has been previously examined, little is known about the underlying mechanisms by which negative workplace behaviors cause their victims to intend to leave. We found evidence in favor of job insecurity as one of the mediators explaining how healthcare personnel's negative workplace behaviors develop and lead to them engaging in turnover intention. The inclination to leave the job rises in healthcare professionals who experience negative workplace behaviors as their perception of job security correspondingly declines. This is consistent with recent meta-analysis research on job insecurity that highlights the multilevel antecedents and consequences of job insecurity (39). Employing the AET theory, job insecurity, as a negative affective experience, is a key point in understanding how events occurring in the work environment affect the behavior of employees. Additionally, the conservation of resource theory states that nurses would devote significant psychological, physiological, and emotional resources to preserving and safeguarding their work, which is inherently important to them if they perceive a threat to the stability or continuity of their employment. But if their efforts are in vain, they will get emotionally weary (18). Negative workplace behaviors, particularly vertical aggression from superiors, can cause medical personnel to worry about job security, future career development, and job rewards when these issues are not handled, they are more likely to quit their job.

Moderation analysis supported the moderation effect of resilience among the negative relationship between negative workplace behaviors and job insecurity. The findings of this study in concert with findings from previous studies demonstrated that medical staff's resilience lessened the detrimental effects of negative workplace behaviors (23). In addition to actively resisting the negative affective experience, healthcare workers who engaged in negative workplace behaviors also actively mobilized their psychological capital and personality resources to counteract the negative affective experience (40). This provided the theoretical foundation for the development of prevention and intervention strategies aimed at halting the detrimental effects of negative workplace behaviors on healthcare workers.

High turnover among healthcare workers impairs patient and worker outcomes and incurs significant financial costs; therefore, nurse managers and organizations should actively investigate factors influencing turnover and develop strategies to prevent turnover. Our study highlighted the adverse outcomes of negative workplace behaviors in the healthcare workplace, including a mechanism to break the vicious circle of high turnover intention among healthcare workers. Mitigating negative workplace behaviors is a key factor in preventing job insecurity, and ultimately reducing the turnover rate of medical staff. Hospital administrator and nurse managers are encouraged to actively engage in the development and enforcement of negative workplace behavior prevention protocols and the building of a harmonious working environment involves the harmonious relationship between colleagues, the superior, and the subordinate. Furthermore, managers should also pay attention to the key role of psychological resilience as a moderator in preventing job insecurity among medical staff, by organizing positive psychology knowledge lectures to help them cultivate confidence and positive attitudes and improve their ability to self-regulate their emotional state.

### 4.1 Limitation

The current study, like all studies, is not free from limitations. First, due to its cross-sectional nature, we were unable to draw causal relationships among negative workplace behaviors, job insecurity, resilience, and turnover intention. To validate and expand upon our findings, longitudinal data are required. Second, self-reports were used to gather all data, a process that incurs unavoidable reporting or recall bias. Future research with more objective data, including hospital turnover rates, might help confirm the results. Third, the participants were recruited from three tertiary hospitals and the sample size was relatively small, which may limit the generalizability of the findings. Future studies should consider broadening the sample's geographical scope.

## 5 Conclusion

Our research revealed that negative workplace behaviors are important factors contributing to employee turnover intention. Job insecurity mediates the relationship between negative workplace behaviors and turnover intention, which indicates reducing healthcare workers' job insecurity is vital to preventing high turnover intention among medical staff. Additionally, psychological resilience moderates the association between negative workplace behaviors and job insecurity.

## Data Availability

The raw data supporting the conclusions of this article will be made available by the authors, without undue reservation.
